# Distribution of quinolone resistance gene (*qnr*) in *ESBL-*producing *Escherichia coli* and *Klebsiella spp.* in Lomé, Togo

**DOI:** 10.1186/s13756-019-0552-0

**Published:** 2019-06-18

**Authors:** Fortune Djimabi Salah, Serge Théophile Soubeiga, Abdoul Karim Ouattara, Adodo Yao Sadji, Amana Metuor-Dabire, Dorcas Obiri-Yeboah, Abiba Banla-Kere, Simplice Karou, Jacques Simpore

**Affiliations:** 1 Biomolecular and Genetic Laboratory (LABIOGENE), Pietro Annigoni Biomolecular Research Center (CERBA), Department of Biochemistry-Microbiology, University Ouaga I Prof Joseph Ki Zerbo, BP 364, Ouagadougou, Burkina Faso; 2 Bacteriology Laboratory, National Institute of Hygiene (INH), BP 1396, Lomé, Togo; 3 High School of Biological and Food Techniques (ESTBA), University of Lomé, BP 1515, Lomé, Togo; 4 Department of Microbiology and Immunology, School of Medical Sciences, University of cape Coast, PMB, Cape Coast, Ghana

**Keywords:** *E. coli*, *Klebsiella spp.*, *ESBL*, *Qnr gene*, Togo

## Abstract

**Background:**

*Qnr* genes are known to confer a low-level resistance to fluoroquinolone in *Enterobacteriaceae*. They are often found on the same resistance plasmids as extended spectrum β-lactamase (ESBL) and constitute the most common antibiotic resistance mechanism. This study aimed to detect the presence of *qn*r genes in *ESBL*-producing *E. coli* and *Klebsiella spp*.

**Methods:**

From May 2013 to July 2015, 91 *E. coli* and 64 *Klebsiella spp.* strains with phenotypic resistance to quinolone were collected from several specimens and analyzed for the detection of *qnrA, qnrB, qnrS* genes and the β-lactamase resistance genes (*blaCTX-M*, *blaTEM*, *blaSHV*) using simplex and multiplex PCR.

**Results:**

In the present study, 107 (69%; 61 *E. coli* and 46 *Klebsiella spp.*) of 155 bacterial strains tested were found harboring at least one *qnr* gene consisting of 74 (47.74%) *qnrB*, 73 (47.10%) *qnrS* and 4 (2.58%) *qnrA*. Of the 107 strains encoding *qnr* genes, 102, 96 and 52 carried *CTX-M1*, *TEM* and *SHV* type ESBL respectively.

**Conclusion:**

This study identified quinolone resistance (*qnr*) gene in *ESBL*-producing *E. coli* and *Klebsiella spp.* in Togo. These finding which suggest a possible resistance to quinolone are of high interest for better management of patients and control of antimicrobial resistance in Togo.

**Electronic supplementary material:**

The online version of this article (10.1186/s13756-019-0552-0) contains supplementary material, which is available to authorized users.

## Background

Quinolones and β-lactams are classes of extensively used molecules worldwide in the treatment of many infectious diseases [1]. Quinolones are synthetic antibiotics used for infections involving Gram-negative bacteria such as *Enterobacteriaceae*. Fluoroquinolones have broad-spectrum intrinsic activity greater than quinolones [2].

Three main mechanisms of quinolone resistance have been described: i) the accumulation of mutations in the genes encoding quinolone target DNA gyrase and topoisomerase IV; ii) a decrease of intracellular concentration of fluoroquinolones by porins down-regulation or modification of the efflux pumps activity, iii) the acquisition of plasmid resistance genes [2].

The acquisition of plasmid-mediated quinolone resistance genes (PMQR) leads to the protection of quinolone’s targets by qnr proteins belonging to the pentapeptide repeat (PRP) family and the hydrolysis of quinolones by the aac (6′)-Ib-cr protein [2, 3]. Mechanism of plasmid-mediated quinolone resistance leads to a low level of fluoroquinolone resistance and facilitates the selection of mutant strains with a high level of fluoroquinolone resistance [3, 4].

Since the discovery of plasmid quinolone resistance genes, a large number of *qnr* alleles have been found on plasmids or bacterial chromosome. About 100 *qnr* genes variant have been described mainly from *Enterobacteriaceae*, and grouped into 5 distinct families: *qnrA*, *qnrB*, *qnrC*, *qnrD* and *qnrS* [3, 5].

Several surveys, based on molecular approaches, have found a strong association between qnr-positive and ESBL-positive isolates [5–8]. The presence of *qnr* genes in ESBL-producing *Enterobacteriaceae* has been reported in Europe, United States, Asia and Africa [9–13]. In Niger, *qnr* genes (9.5% of *qnrA*, 26.2% of *qnrB* and 64.3% of *qnrS*) were found in ESBL-producing *Enterobacteriaceae* among fecal commensal of children with severe malnutrition [14].

In Togo, results from previous studies revealed the presence of beta-lactamase gene *CTX-M1* (95.73%), *TEM* (82.31%) and *SHV* (45.12%) in ESBL-producing *E. coli* and *Klebsiella spp.* The production of ESBL was associated with high co-resistance to fluoroquinolone (93% for ciprofloxacin), aminoglycosides (76.36% for gentamicin) and trimethoprim/sulfamethoxazole (95.65%) [15, 16]. This high level of multidrug resistance suggests acquisition of plasmid-mediated antibiotic resistance factors in these strains. PMQR determinants as *qnr* genes was also usually found in multidrug resistance plasmid among *Enterobacteriaceae* producing-ESBL especially *E. coli* and species of *Klebsiella* [3, 6, 17, 18]. In this study we are interested in fluoroquinolone resistance. Here, we report the frequency of *qnrA*, *qnrB* and *qnrS* genes in ESBL-producing *E. coli* and *Klebsiella spp.*

## Methods

### Samples collection and identification

Well characterized *Escherichia coli* and *Klebsiella spp.* strains were collected during a prospective study from May 2013 to July 2015 in the bacteriology laboratory of the National Institute of Hygiene (INH) in Lomé, Togo. This public health institute is specialized in biomedical analysis, epidemiological surveillance, immunization, water, and food quality control. Strains were isolated from various pathological specimens including urine, vaginal swabs, pus, and sperm samples. Standard microbiological methods were used to isolate and purify bacterial strains on Mac-Conkey or Eosin Methylene Blue (EMB) media. Strains were identified using the API 20E identification system (API 20 E, Identification System for Enterobacteriaceae and others non-fastidious Gram-negative rod; BioMérieux, Marcy-Etoile, France). The API 20 E system is a standardized technique allowing only the biochemical identification of an Enterobacterial strain using an isolated colony.

### Susceptibility test and ESBL phenotype detection

Antibiotic susceptibility test was performed and interpreted according to the 2014 recommendations of Antibiogram Committee of the French Society of Microbiology [19].

Antibiotics were purchased from BioRad (Marnes-la-Coquette, France) and included amoxicillin + clavulanate (AMC, 20/10 μg), piperacillin-tazobactam (TZP, 75/10 μg), cefoxitin (FOX, 30 μg), ceftriaxon (CRO, 30 μg), ceftazidim (CAZ, 30 μg), cefotaxim (CTX, 30 μg), cefepim (FEP, 30 μg), aztreonam (ATM, 30 μg), imipenem (IPM, 10 μg), amikacin (AKN, 30 μg), gentamicin (G, 15 μg), nalidixic acid (NA, 30 μg) ciprofloxacin (CIP, 5 μg), trimethoprim-sulfamethoxazole (SXT 1.25/23,75 μg), fosfomycin (FOS, 50 μg), doxycycline (DOX, 30 μg).

All isolates were subjected to the double disc synergy test for ESBL detection [20]. The presence of ESBL is detected by a synergy between ceftazidim and cefotaxim or ceftriaxon discs and amoxicillin + clavulanic acid disc.

*E. coli* ATCC 25922 strain was used as a control for antibiotic susceptibility testing.

*Escherichia coli* and *Klebsiella spp.* strains resistant to at least one third generation cephalosporin (cephalosporin, ceftazidim, ceftriaxon or cefotaxim) were collected in a storage medium (trypticase soy broth TCS) and stored at − 80 °C. Samples were then sent under strict transportation conditions (in triple packaging boxes with ice packs), to Molecular Biology Laboratory of CERBA/LABIOGENE in Ouagadougou, Burkina Faso for *qnr* and *ESBL* genes detection on in January 2018.

### Extraction of bacterial DNA

Rapid DNA extraction was performed using a boiling technique. Shortly, strains from TCS broth were reactivated on TCS agar for 18–24 h and two or three isolated colonies were inoculated in Luria Bertani (LB, 2 mL). After 18–24 h of overnight culture, LB broth were centrifuged at 10000 rpm/min for 10 min and the pellet suspended in 500 μL of phosphate buffer (100 mM, pH 7) to cell-wall weakening. The mix was heated at 100 °C for 15 min in a water bath to release bacterial nucleic acid.

DNA was then precipitated in 250 μL of absolute ethanol, washed twice in 1000 μL of ethanol 75%, dried and re-suspended in 100 μL of sterile water.

### PCR amplification

DNA samples (5 μL) were subjected to multiplex PCR in a 25 μL reaction mixture as previously described by Robicsek [21] for *Qnr* genes and Dallenne [22] for *ESBL* genes *blaTEM* and *blaSHV* using GeneAmp® PCR System 9700 (Applied Biosystems, California USA).

*Qnr* genes (*qnrA*, *qnrB* and *qnrS*) amplification was performed using the following thermal cycling profile: 32 cycles consisting of 45 s at 95 °C for denaturation, 45 s at 53 °C for annealing and 60 s at 72 °C for extension.

For the *blaTEM* and *blaSHV ESBL* genes, multiplex PCR amplification conditions were as follows: initial denaturation step at 94 °C for 10 min; 30 cycles of denaturation at 94 °C for 40s, annealing at 60 °C for 40s, extension at 72 °C for 1 min, followed by a final extension step at 72 °C for 7 min. However, the amplification of *BlaCTX-M-G1* was carried out as previously described by Pagani [23] in 25 μL reaction mixture according to the following PCR program: initial denaturation at 96 °C for 10 min. 35 cycles of denaturation at 94 °C for 1 min, annealing at 50 °C for 1 min and extension at 72 °C for 1 min. Final extension at 72 °C for 10 min. Negative (DNA from *E. coli* ATCC 25922) and positive controls (DNA from *qnr B* and *S* genes positive strains) were used to check potential unspecific amplification. Specific sequences primers provided by Applied Biosystems (California, USA) are shown in Table 1. DNA fragments were analyzed by electrophoresis in a 2% agarose gel at 100 V for 1 h in TBE 1X containing ethidium bromide using 100-bp DNA ladder (Promega, USA) as a size marker.

**Table 1:** Primers used for PCR amplification of qnr and bla genes identification

*Bla* Genes	Sequence (5′ – 3′)	Size (pb)	References
*qnrA*	For: ATTTCTCACGCCAGGATTTG Rev.: GATCGGCAAAGGTTAGGTCA	516	[21]
*qnrB*	For: GATCGTGAAAGCCAGAAAGG Rev.: ACGATGCCTGGTAGTTGTCC	469	[21]
*qnrS*	For: ACGACATTCGTCAACTGCAA Rev.: TAAATTGGCACCCTGTAGGC	417	[21]
*TEM*	For: CATTTCCGTGTCGCCCTTATTC Rev.: CGTTCATCCATAGTTGCCTGAC	800	[22]
*SHV*	For: AGCCGCTTGAGCAAATTAAAC Rev.: ATCCCGCAGATAAATCACCAC	713	[22]
*CTX-M-G1*	For: GTTACAATGTGTGAGAAGCAG Rev.: CCGTTTCCGCTATTACAAAC	1000	[23]

### Statistical analysis

Statistical analysis was performed using Epi Info Version 7.1.1.14 software. Fisher’s exact test was used for comparison and the difference was statistically significant when *p* < 0.05.

## Results

### Bacterial strains

A sample of 155 strains, 91 *E. coli* and 64 *Klebsiella spp.* (55 *Klebsiella pneumoniae* and 9 *Klebsiella oxytoca*) resistant to at least one third generation cephalosporin (ceftazidim, cefotaxim or ceftriaxon) were collected during the study period. Bacteria were isolated from urine 91/155 (58.71%), vaginal samples 38/155 (24.52%), wound swabs 15/155 (9.69%), semen samples 6/155 (3.87%), urethral curettage 2/155 (1.29%), sputum 1/155 (0.65%), stool 1/155 (0.65%) and joint fluid 1/155 (0.65%).

### Antibiotic susceptibility profile

All *E. coli* strains were resistant to ceftriaxon and cefotaxim and 97.80% to ceftazidim. The resistance rates to other β-lactam antibiotics were 2.20% for imipenem (very low levels), 17.59% for piperacillin-tazobactam and 25.35% for cefoxitin. Quinolones, nalidixic acid and ciprofloxacin were very inactive with rates of 96.67% and 94.51% respectively. Among aminoglycosides, the strains were more resistant to gentamicin (75.82%) in contrast to amikacin which showed very low levels of resistance (3.30%).

All *Klebsiella spp.* strains were resistant to cefepim. Resistance to ceftazidime and ceftriaxone was 98.44% (63/64) each one, and to cefotaxime 96.88% (62/64). Nalidixic acid, ciprofloxacin, doxycycline and trimethoprim-sulfamethoxazole were also inactive antibiotics with at least a resistance rate of 90%. Only imipenem, amikacin and fosfomycin were very active on *Klebsiella spp.* strains with a low resistance rate (< 5%). The resistance profile to other beta-lactams and other antibiotics for all isolates is presented in Table 2.

**Table 2:** Antibiotic susceptibility profile

ATB	*E. coli*		*Klebsiella spp*		Total	
	R (n/N)	R (%)	R (n/N)	R (%)	R (n/N)	R (%)
TZP	16/91	17.58	19/24	29.69	35/155	22.58
FOX	18/71	25.35	12/52	23.08	30/123	24.39
CAZ	89/91	97.80	63/64	98.44	152/155	98.06
CRO	91/91	100	63/64	98.44	154/155	99.35
CTX	91/91	100	62/64	96.88	153/155	98.71
FEP	89/91	97.80	64/64	100	153/155	98.71
ATM	90/91	98.90	63/64	98.44	153/155	98.71
IMP	2/91	2.20	1/64	1.56	3/155	1.94
G	69/91	75.82	51/64	79.69	120/155	77.42
AKN	3/91	3.30	1/64	1.56	4/155	2.58
NA	87/90	96.67	51/63	80.95	138/155	90.20
CIP	86/91	94.51	58/64	90.63	144/155	92.90
FOS	4/90	4.44	3/64	4.69	7/154	4.55
SXT	85/90	94.44	56/61	96.72	144/151	95.36
DOX	86/89	96.63	58/63	92.06	144/152	94.74

*ATB* antibiotic, *R* resistant, *TZP* piperacillin-tazobactam, *FOX* cefoxitin, *CAZ* ceftazidim, *CRO* ceftriaxon, *CTX* cefotaxim, *FEP* cefepim, *ATM* aztreonam, *IPM* imipenem, *G* gentamicin, *AKN* amikacin, *NA* nalidixic acid, *CIP* ciprofloxacin, *FOS* fosfomycin, *SXT* trimethoprim / sulfamethoxazole

The ESBL were phenotypically detected in 87/91 (95.60%) *E. coli* and 62/64 (96.88%) *Klebsiella spp.* strains.

### Distribution of *qnr* genes

**Fig. 1:**
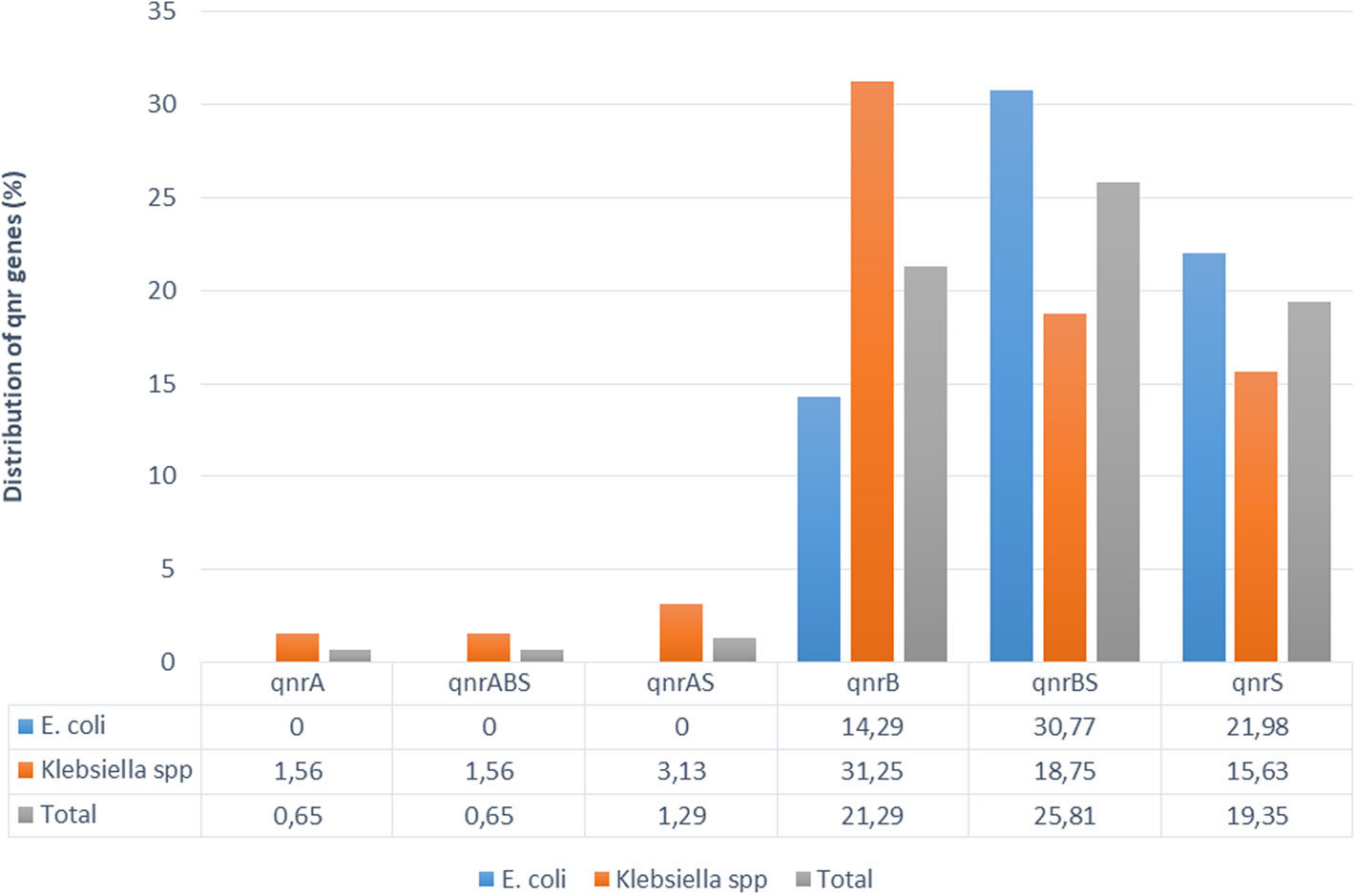
Distribution of *qnr* genes in bacterial species

Electrophoresis analysis revealed 107 strains (61 *E. coli* and 46 *Klebsiella spp.*) harboring at least one *qnr* gene: 74 (47.74%) *qnrB* (41 *E. coli* and 33 *Klebsiella spp.*), 73 (47.10%) *qnrS* (48 *E. coli* and 25 *Klebsiella spp.*) and 4 (2.58%) *qnrA* (*Klebsiella spp.* only). However, any *qnr* genes were not detected in 48 strains. The concomitant presence of two or three *qnr* genes was detected. An additional figure showed agarose gel electrophoresis in more detail (see Additional file 1). Proportions of *qnrBS* combinations were 30.77% and 18.75% respectively in *E. coli* and *Klebsiella spp.*; *qnrAS* was observed in 3.13% of *Klebsiella spp.* while the triple association *qnrABS* was found in one *Klebsiella spp.* strains*. QnrA* was not found in *E. coli* strains. The prevalence of *qnr* genes was higher in *Klebsiella spp*. 71.88% (46/64) compared to *E. coli* 67.03% (61/91) strains. Distribution of *qnr* genes in bacterial species is shown in Fig. 1.

### Antibiotic susceptibility profile of qnr-positive strains

Out of the 107 (61 *E. coli* + 46 *Klebsiella spp.*) strains encoding qnr genes, 95/105 (90.48%) were resistant to nalidixic acid (58 *E. coli* + 37 *Klebsiella spp.*) and 100/107 (93.46%) were resistant to ciprofloxacin (57 *E. coli* + 43 *Klebsiella spp.*); 105/107 (98.13%) resistant to ceftazidim (59 *E. coli* + 46 *Klebsiella spp.*) and 106/107 (99.07%) to ceftriaxon (61 *E. coli* + 45 *Klebsiella spp.*). The resistance rates to gentamicin were 82.24% (88/107).

These strains remained however susceptible to imipenem (97.20%), amikacin (97.20%), and fosfomycin (95.33%). The resistance profile is presented in Table 3.

**Table 3:** Antibiotic susceptibility profile of *qnr* positive strains

ATB		*E. coli*	*Klebsiella spp.*	Total	
				n/N	%
NA	R	58/60	37/45	95/105	90.48
	S	2/60	8/45	10/105	9.52
CIP	R	57/61	43/46	100/107	93.46
	S	4/61	3/46	7/107	6.54
CAZ	R	59/61	46/46	105/107	98.13
	S	2/61	0/46	2/107	1.87
CRO	R	61/61	45/46	106/107	99.07
	S	0/61	1/46	1/107	0.93
IMP	R	2/61	1/46	3/107	2.80
	S	59/61	45/46	104/107	97.20
AKN	R	2/61	1/46	3/107	2.80
	S	59/61	45/46	104/107	97.20
G	R	48/61	40/46	88/107	82.24
	S	13/61	6/46	19/107	17.76
FOS	R	4/61	1/46	5/107	4.67
	S	57/61	45/46	102/107	95.33

*R* resistant, *S* sensible, *CAZ* ceftazidim, *CRO* ceftriaxon, *IPM* imipenem, *G* gentamicin, *AKN* amikacin, *NA* nalidixic acid, *CIP* ciprofloxacin, *FOS* fosfomycin

Most isolates that were resistant to ciprofloxacin and nalidixic acid encoded *qnrB* and *qnrS* alone or in association but no *qnr* genes was detected in 29 *E. coli* and 15 *Klebsiella spp.* strains resistant to ciprofloxacin and nalidixic acid. Nevertheless, isolates encoding *qnrB* or *qnrS* were also identified among nalidixic acid-susceptible (10/105) and ciprofloxacin-susceptible strains (7/107) (Table 4).

**Table 4:** Co-existence of qnr gene and bla gene in *E. coli* and *Klebsiella spp.*

Species		E. coli (*N* = 61)				Klebsiella spp (*N* = 46)						
Qnr gene		*qnrB* (*n* = 13)	*qnrBS* (*n* = 28)	*qnrS* (*n* = 20)	Total 1	*qnrA* (*n* = 1)	*qnrABS* (*n* = 1)	*qnrAS* (*n* = 2)	*qnrB* (*n* = 20)	*qnrBS* (*n* = 12)	*qnrS* (*n* = 10)	Total 2
NA	R	12	28	18	58	1	1	2	17	10	6	37
	S	1	0	1	2	0	0	0	2	2	4	8
CIP	R	11	28	18	57	1	1	2	20	11	8	43
	S	2	0	2	4	0	0	0	0	1	2	3
ESBL genes	CTX-M1	2	4	1	7	0	0	0	0	0	0	0
	SHV CTX-M1	0	0	0	0	0	0	0	0	3	1	4
	TEM	0	0	1	1	0	0	0	0	0	0	0
	TEM CTX-M1	6	16	14	36	0	1	0	5	2	3	11
	TEM SHV	0	0	1	1	0	0	0	1	2	0	3
	TEM SHV CTX-M1	5	8	3	16	1	0	2	14	5	6	28

*R* resistant, *S* sensible, *NA* nalidixic acid, *CIP* ciprofloxacin, *ESBL* extended spectrum β-lactamase, *qnr* quinolone resistance

### Distribution of qnr genes among ESBL-producing isolates

PCR was performed to determine the presence of *ESBL* genes and all strains, positive or not to the double disc synergy test, carried at least one genes *blaTEM*, *blaSHV* and/or *blaCTX-M1*. All *E. coli* and *Klebsiella spp.* strains *qnr* positive were ESBL-producing. Within the 107 strains encoding *qnr* genes (61 *E. coli* + 46 *Klebsiella spp.*), 102 carried *CTX-M1* (59 *E. coli* + 43 *Klebsiella spp.*), 96 carried *TEM* (54 *E. coli* + 42 *Klebsiella spp.*) and 52 carried *SHV* (17 *E. coli* + 35 *Klebsiella spp.*).

Results revealed that *qnr* subtypes (*A, B, S*) could co-exist alone or in association with *blaCTX-M1*, *blaTEM* and *blaSHV*. Among *E. coli* strains, *qnrBS* combinations was most frequently associated with *TEM/CTX-M1* combinations and among *Klebsiella spp.*, the most frequent association was *qnrB* plus *TEM/SHV/CTX-M1* (Table 4).

## Discussion

Plasmid-mediated quinolone resistance may facilitate the spread and increase frequency of quinolone-resistant strains. Until now *qnr* genes have been widely detected in different parts of the world. Such data are not available in Togo. This is the first study which reports the frequency and diversity of *qnr* genes among ESBL-producing *Enterobacteriaceae* in Togo.

The highest rate was found among *Klebsiella spp.* (71.88%) and *E. coli* (67.03%). Three *qnr* groups were detected and are described in this report. Among all the isolates detected, *qnrB* (47.74%) and *qnrS* (47.10%) were the most predominant, followed by *qnrA* (2.58%).

These frequencies found in this study are higher than those reported in Côte d’Ivoire where *qnr* genes were found at 31.2% in *E. coli* and 20.5% in *Klebsiella spp*. with 14.6% for *qnrB*, 9.9% for *qnrA* and 2.7% for *qnrAS* [10]. Always in Côte d’Ivoire, others authors found 50.54% of *qnr* genes in *Klebsiella pneumoniae* (71.73% *qnrB*, 26.08% *qnrS* and 2.17% *qnrA*) [24].

In Niger, *qnr* genes were also reported at 93.3% in *Klebsiella spp*. and 44.4% in *E. coli* with 64.3% for *qnrS*, 26.2% for *qnrB* and 9.5% for *qnrA* [14].

In Morocco, *qnrB* was found at 23%, *qnr A* at 10% and *qnrS* at 3% in 50% *Klebsiella spp.* and 18.7% *E. coli* [9]. In 2014 in Moroccan community enterobacteria, the prevalence of *qnr* gene was 2.6% (1.7% *qnrS1* and 0.9% *qnrB*) [25]. These genes are usually plasmid mediated and can easily spread among the members of *Enterobacteriaceae*, through gene transfer mechanisms [3, 6, 17, 18]. Results of plasmid isolation test and conjugation experiments in different studies indicated that these *qnr* gene were carried by conjugative plasmid of high molecular weight. These determinants can be transferred between bacteria, thus realizing the epidemic spread of quinolone resistance through horizontal gene transfer [11, 13, 25–28]. However, due to the lack of financial resources, conjugation experiments or hybridization confirming the presence of target genes on plasmids were not performed in the present study.

Among strains encoding the *qnr* gene in our study, 90.48% were resistant to nalidixic acid, 93.46% to ciprofloxacin, 98.13% to ceftazidim and 99.07% to ceftriaxon. These rates are higher than those observed in Morocco (57% for nalidixic acid and 78% ciprofloxacin, 100% for ceftazidim and 71% for cefotaxim) [9]. In Mexico, the resistance rate among *qnr* positive pediatric strains was 41.1% for nalidixic acid, 29.4% for ciprofloxacin, 82.3% for ceftazidim and 100% for cefotaxim [13]. In this study, higher resistance rate of the *qnr* positive strains against nalidixic acid and ciprofloxacin could be explained by the concomitant presence of two or three *qnr* gene groups (43/107, 40.20%), also found in Algeria and in Vietnam, thus inducing an additive effect on the minimal inhibiting concentration (MICs) of these different molecules. In addition, *qnr* positive isolates showed more resistance to gentamicin (82.24%). This may be explained by the fact that plasmid-mediated quinolone resistance is associated with integrons bearing resistance determinants to several other antibiotics such as beta-lactams and aminoglycosides [3, 4, 27, 29]. The *qnr* genes were identified among isolates which were susceptible to nalidixic acid and ciprofloxacin.

This result has clinical implications since the acquisition of the *qnr* genes by quinolone susceptible ESBL-producing strains could lead to selection of ciprofloxacin and cephalosporin resistant strains an increasing the mutant prevention concentration (MPC) [6, 17]. However no *qnr* genes was detected in 29 *E. coli* and 15 *Klebsiella spp.* strains resistant to ciprofloxacin and nalidixic acid, can suggest the presence of another mechanism of resistance to quinolones such as mutations in the gyrase and topoisomerase IV genes [2, 6, 30].

The presence of *ESBL* and some of the quinolone-resistant genes in the same mobile genetic elements could explain the co-resistance to beta-lactams and fluoroquinolones. Our results showed that all *E. coli* and *Klebsiella spp.* strains *qnr* positive were *ESBL*-producing. Among *qnr*-positive strains, 102 produce *CTX-M1*, 52 *SHV* and 96 produce *TEM*.

Previous studies showed that *qnr*-positive strains frequently expressed *ESBL* [9–11, 28, 31, 32]. The strong association between PMQR gene and *blaCTX-M-15* and *blaTEM-116* was detected in clinical Enterobacterial isolates from Iran [31]. In Mexico, characterization of adult *qnr*-positive isolates indicated that the SHV ESBL-type (SHV-12, − 5, and 2a) was the most prevalent (81.6%) followed by CTX-M-15 (44.9%) [28]; but in pediatric isolates, CTX-M-15 was the most predominant (70.5%) [13]. However, in both bacterial population, combination of *ESBL* and *qnr* genes may be pointing to a co-selection of cephalosporin and quinolone resistance. *QnrA1* and *qnrS1* have previously been found to be associated with *blaCTX-M-9*, *blaSHV-12* and *blaSHV-92* among Enterobacterial isolates in Spain [11]. *QnrB* was observed to be co-produced with CTX-M-15 in Algeria strains of *E. coli* [33]. A similar result was recently found in *Klebsiella pneumoniae* isolates from Côte d’Ivoire, but the type of ESBL was not determined [24]. *Qnr* genes are usually found in multi-resistance plasmids linked to other resistance determinants, beta-lactamase genes have been conspicuously common [5, 6, 25, 26].

Our results also revealed that *qnr* subtypes could co-exist alone or in association with *beta-lactamase* genes.

Among *E. coli* strains, *qnrBS* combinations was most frequently associated with *blaTEM/CTX-M1* combinations and among *Klebsiella spp.*, the most frequent association was *qnrB* plus *blaTEM/SHV/CTX-M1*. The *blaCTX-M1*, plasmid-mediated class A *ESBL* [34–36] expression was observed to be currently more frequent in a double or triple combination with *blaTEM* and *blaSHV*. This genes combination suggests a progressive consolidation of resistance genes on a single mobile genetic element (plasmids, integrons, etc.).

These findings raise the hypothesis that the *qn*r genes detected in these strains could also have the same plasmid location. Plasmid isolation and conjugation experiments should be then investigated to confirm the presence of target genes on plasmids. Our results also suggested the community emergence of *PMQR* determinants (*qnr gene*) that contributed to the development and spread of fluoroquinolone resistance in *E. coli* and *Klebsiella spp.* isolates in Togo. The presence of these determinants in the outpatient is worrisome, due of the potential spread of plasmids in a scenario of uncontrolled oral quinolone usage, which can compromise therapeutic options and therefore concern for public health.

The potential limitations of this study were the absence of data on minimal inhibiting concentration (MIC) for nalidixic acid and ciprofloxacin to determine the level of bacterial resistance to these antibiotics and also the bias of including only *ESBL* strains and the absence of molecular typing of strains and sequence analysis of the different genes.

Altogether, the results of the present study underline the frequency of *qnr* determinants associated to fluoroquinolones resistance among *E. coli* and *Klebsiella spp* ESBL-producing strains in Togo and identifies the presence of *qnr* genes in quinolone-susceptible strains which could lead to in vivo selection of ciprofloxacin-resistant strains.

## Conclusion

This first report of *qnrA*, *qnrB* and *qnrS* gene among *ESBL*-producing *E. coli* and *Klebsiella spp.* from Togo, extends upon similar finding in many countries supporting the wide distribution of *qnr* genes.

The results revealed a high rate of *qnrB* and *qnrS* alone or in combination and a higher association with *blaTEM/CTX-M1* and *blaTEM/SHV/CTX-M1* combinations. These *qnr* genes positive strains were highly resistant to nalidixic acid, ciprofloxacin, ceftazidim, ceftriaxon and gentamycin. However, they remain susceptible to imipenem, amikacin and fosfomycin.

The plasmid-mediated quinolone resistance genes and their association with cephalosporin resistance mediated by *ESBL* contribute to the spread of multidrug resistance due to their easy transfer between bacteria. Their wide dissemination impairs treatment outcome of common infections in community and hospitals settings. These finding suggest the strengthening of the public health policies in Togo in order to prevent, monitor and control antimicrobial resistance through the implementation of an antibiotic resistance surveillance system. Further studies on sequence analysis of the *ESBL* gene amplicons are also needed to determine different resistance profile of *ESBL*-producing bacteria in Togo.

## Additional file

Additional file 1: Agarose gel electrophoresis (2%) used for the separation of multiplex PCR products. M: molecular size marker (100 bp ladder, Promega, USA); line 1, 8, 9, 12: negative; line 2, 4, 5, 7, 10, 11, 14, 15, 17, 18, 19, 20: qnr B + qnr S genes: line 3: qnr A + qnrB + qnr S genes; line 6: qnrS genes; line 13: qnrB genes; line 21: negative control and line 22: positive control qnrB genes. Qnr A (517 bp), qnrB (469 bp), qnr S (417 bp). (PDF 325 kb)

## Data Availability

The database analyzed during the study is available on reasonable request from the corresponding author.

## Abbreviations

AKN: Amikacin
AMC: Amoxicillin + clavulanate
ATCC: American Type Culture Collection
ATM: Aztreonam
CASFM/EUCAST: Comité d’Antibiogramme de la Socièté Française de Microbiologie/EUropean Committee on Antimicrobial Susceptibility Testing
CAZ: Ceftazidim
CERBA: Centre de Recherche Biomoléculaire Pietro Annigoni
CIP: Ciprofloxacin
CRO: Ceftriaxon
CTX: Cefotaxim
CTX-M: Cefotaximase-Munich
DNA: Desoxyribonucleic Acid
DOX: Doxycyclin
EMB: Eosin Methylene Blue
ESBL: Extended Spectrum Beta-Lactamase
ESTBA: Ecole Supérieure des Techniques Biologique et Alimentaire
FEP: Cefepim
FOS: Fosfomycin
FOX: Cefoxitin
G: Gentamycin
IMP: Imipenem
INH: Institut National d’Hygiène
LABIOGENE: Laboratoire de Biologie Moléculaire et de Génétique Moléculaire
NA: Nalidixic acid
PMQR: Plasmid Mediated Quinolone Resistance
PRP: Pentapeptid Repeat Protein
*qnr*: quinolone resistance
SHV: Sulfhydryl Variable
SXT: trimethoprim/sulfamethoxazole
TCS: Trypticase Soy
TEM: Temoniera
TZP: Piperacillin-tazobactam
