# Supplementary material for: Distribution of quinolone resistance gene (qnr) in ESBL-producing Escherichia coli and Klebsiella spp. in Lomé, Togo
Source: Antimicrob Resist Infect Control. 2019 Jun 18;8:104. doi: 10.1186/s13756-019-0552-0 (PMC6582466; doi:10.1186/s13756-019-0552-0)
Supplement: Supplementary file 1 — Agarose gel electrophoresis (2%) used for the separation of multiplex PCR products. M: molecular size marker (100 bp ladder, Promega, USA); line 1, 8, 9, 12: negative; line 2, 4, 5, 7, 10, 11, 14, 15, 17, 18, 19, 20: qnr B + qnr S genes: line 3: qnr A + qnrB + qnr S genes; line 6: qnrS genes; line 13: qnrB genes; line 21: negative control and line 22: positive control qnrB genes. Qnr A (517 bp), qnrB (469 bp), qnr S (417 bp). (PDF 325 kb) [file 13756_2019_552_MOESM1_ESM.pdf]

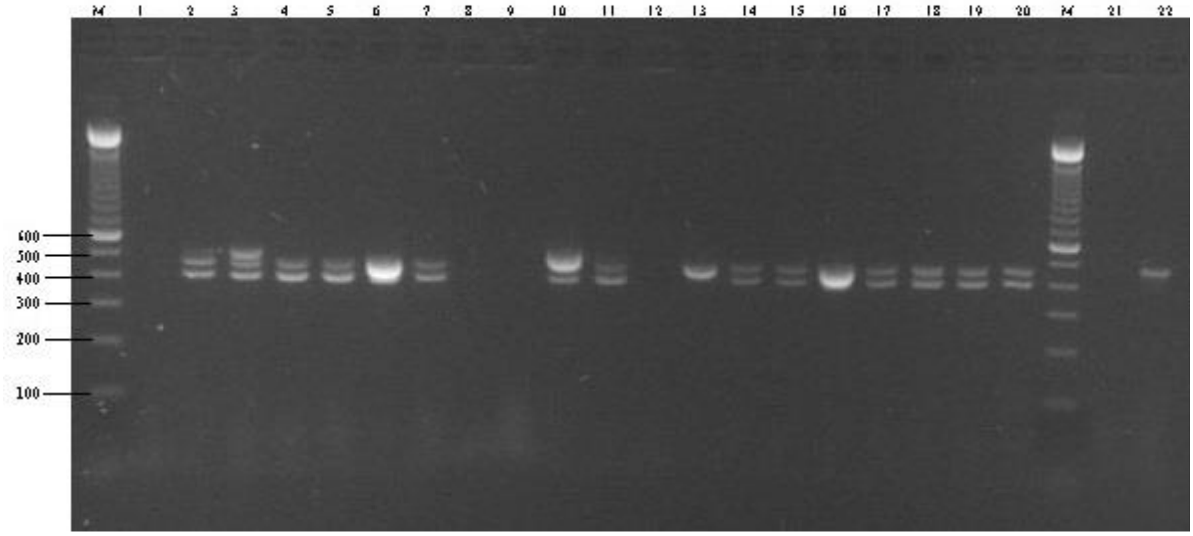

Figure 2: Agarose gel electrophoresis (2%) used for the separation of multiplex PCR products. M: molecular size marker (100 bp ladder, Promega, USA); line 1, 8, 9, 12: negative; line 2, 4, 5, 7, 10, 11, 14, 15, 17, 18, 19, 20: *qnr B* + *qnr S* genes: line 3: *qnr A* + *qnrB* + *qnr S* genes; line 6: *qnrS* genes; line 13: *qnrB* genes; line 21: negative control and line 22: positive control *qnrB* genes. *Qnr A* (517 bp), *qnrB* (469 bp), *qnr S* (417 bp).
